# Optimization Design of a Multi-String Standing Wave Electrospinning Apparatus Based on Electric Field Simulations

**DOI:** 10.3390/polym16162330

**Published:** 2024-08-17

**Authors:** Xiaoqing Chen, Jiahao Liang, Xiang Tan, Jiazheng Ding, Wenyu Xie, Changgang Li, Yebin Cai

**Affiliations:** 1 School of Energy and Power Engineering, Guangdong University of Petrochemical Technology, Maoming 525099, China; chenxiaoqing0710@163.com (X.C.); mmcyb@126.com (Y.C.); 2 Key Laboratory of Petrochemical Pollution Control of Guangdong Higher Education Institutes, Guangdong Engineering Technology Research Center of Petrochemical Pollution Control, School of Environmental Science and Engineering, Guangdong University of Petrochemical Technology, Maoming 525099, Chinadingjz1999@163.com (J.D.); licg0208@163.com (C.L.); 3 Institute of Science and Environment, University of Saint Joseph, Macau 999078, China; 4 State Key Laboratory of Petroleum Pollution Control, China University of Petroleum-Beijing, Beijing 102249, China

**Keywords:** needleless electrospinning, polymer nanofiber, electric field simulations, standing wave electrospinning

## Abstract

The mass production of uniform, high-quality polymer nanofibers remains a challenge. To enhance spinning yield, a multi-string standing wave electrospinning apparatus was developed by incorporating a string array into a standing wave electrospinning device. The process parameters such as string spacing, quantity, and phase difference were optimized, and their effects on the electric field distribution within the spinning area were analyzed using electric field simulations. When the string spacing was less than 40 mm or the number of strings exceeded two, the electric field strength significantly decreased due to electric field interference. However, this interference could be effectively mitigated by setting the string standing wave phase difference to half a period. The optimal string array parameters were identified as string spacing of 40 mm, two strings, and a phase difference of half a period. Multi-string standing wave electrospinning produced fibers with diameters similar to those obtained with single-string standing wave electrospinning (178 ± 72 nm vs. 173 ± 48 nm), but the yield increased by 88.7%, reaching 2.17 g/h, thereby demonstrating the potential for the large-scale production of nanofibers. This work further refined the standing wave electrospinning process and provided valuable insights for optimizing wire-type electrospinning processes.

## 1. Introduction

Nanofibers possess unique characteristics, such as high porosity, a large specific surface area, and remarkable scale and surface effects [1]. These attributes make them highly desirable for various applications, including biomedicine [2,3], energy storage [4,5], sensors [6,7,8], environmental remediation [9], and personal protection [10]. The demand for high-quality nanofibers is increasing across numerous industries, emphasizing the need for improved spinning techniques.

Electrospinning is an efficient and novel technical way of generating continuous nanofibers with diameters between ten nanometers and several micrometers [11]. It uses a high-voltage DC electric field that causes the droplets of a polymer solution to undergo Taylor cone deformation and stretching, ultimately forming fibers [12]. Electrospinning is considered the simplest and most industrially viable technology for fabricating polymer nanofibers because of its simple device structure, wide applicability, and low operating costs [13,14]. Single-needle electrospinning is the most traditional electrospinning technique; however, its fiber production rate is significantly low, averaging only about 0.01–0.1 g/h [15]. The development of multi-needle capillary array electrospinning, which utilizes an array of multiple needles, has increased the fiber production rate [16]. However, issues such as the difficulty in cleaning and clogging the spinning needles, as well as the decrease in fiber quality due to electric field interference, remain unresolved [17,18]. Needleless electrospinning forms Taylor cones and jets through the self-organization of the spinning liquid, which fundamentally solves the blockage problem of needle spinning and shows a higher fiber productivity [18,19]. Current studies have proposed bubble-electrospinning [20], conical wire electrospinning [21], multiple parallel electrode electrospinning [22], and curved convex slot electrospinning [23], among others. However, most require high spinning voltages (>30 kV), and the fiber uniformity and quality need further improvement.

In our previous research, we developed a standing wave electrospinning device that utilized string standing wave vibrations to enhance Taylor cone formation. This innovation notably reduced the spinning threshold voltage to 18 kV and successfully fabricated nanofibers with a diameter of 173 ± 48 nm [24]. However, the production of single-string electrospinning was limited and often insufficient to meet the demand for mass production. Constructing string arrays was widely regarded as an efficient way to increase spinning production. However, there were still few reports detailing the specific enhancement effects and whether this method impacts fiber quality. How simultaneous spinning with multiple strings affects the electric field intensity in the spinning area remained unclear. The optimal spacing between the strings to avoid electrostatic interference also needed to be determined. Additionally, the potential for the phase difference in standing waves on the strings to reduce electric field interference required further investigation. 

In this study, we constructed a multi-string standing wave electrospinning apparatus by installing string arrays in the standing wave electrospinning device. The process parameters, including string spacing, quantity, and phase difference, were optimized, and their effects on the electric field distribution within the spinning area were investigated using electric field simulations. Additionally, the fiber quality and production of the multi-string standing wave electrospinning were further evaluated. This work proposes new solutions for the mass production of high-quality fibers and provides references for optimizing wire-type electrospinning processes.

## 2. Materials and Methods

### 2.1. Materials

Polyvinyl alcohol (PVA1799, Mw = 72,600–81,400) and sodium dodecyl sulfate (C_12_H_25_SO_4_Na) were purchased from Chenqi Chemical Technology Company (Shanghai, China). Guitar strings made of phosphor bronze were purchased from Guangzhou Romance Musical Instrument Manufacturing Co., Ltd. (Guangzhou, China). The diameter and conductivity of the strings were 1 mm and 5.8 × 10^7^ S/m, respectively.

### 2.2. Preparation of PVA Solution

PVA powders (50 g) were swelled in 500 mL of sodium dodecyl sulfate solution (0.2 wt%) at 25 °C for 30 min. The swollen PVA was subsequently dissolved in 500 mL of deionized water and stirred at 220 r/min at 90 °C for 3 h. The PVA solution was stored at 4 °C in the refrigerator before use.

### 2.3. Multi-String Standing Wave Electrospinning Apparatus

**Figure 1:**
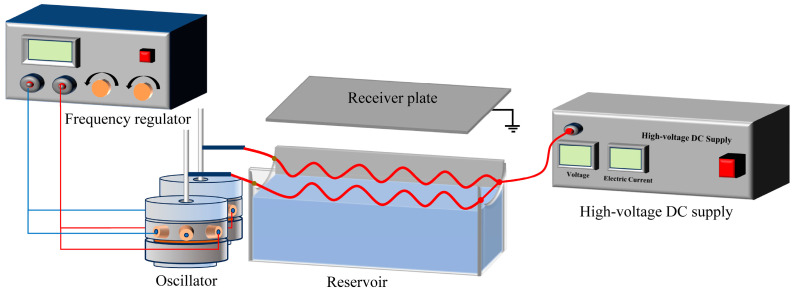
The schematic diagram of a multi-string standing wave electrospinning apparatus.

The multi-string standing wave electrospinning apparatus comprised a frequency regulator, oscillator, string array, solution reservoir, high-voltage DC supply, and receiver plate (Figure 1). One end of each string was connected to the high-voltage DC supply, and the other end was connected to the oscillator. The 1 mm thick receiver plate made of bronze was positioned vertically above the solution reservoir. The oscillator was employed to control the amplitude and number of standing waves on the string by adjusting the oscillation amplitude and vibration frequency of the oscillator head. The frequency regulator governed the vibration frequency. The spinning parameters were set according to the optimal conditions of the single-string standing wave electrospinning apparatus, as previously described by Chen et al. [24]. The applied voltage, spinning distance, and standing wave number were set at 28 kV, 10 cm, and 3, respectively. The effective length of the string was 60 cm. The spinning process is shown in Figure S1.

### 2.4. Electric Field Simulation

**Figure 2:**
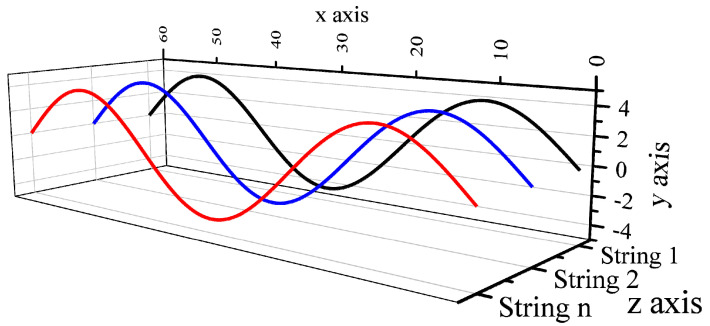
The arrangement of string arrays.

String vibration was fitted to the mathematical model of standing waves [24]. The equation and boundary conditions were described as

(1) $$\left\{\begin{array}{l} y=2A\sin\left(\frac{\pi n}{l}x\right)\cos\left(2\pi ft\right)\\ y\left(l,t\right)=0 \end{array}\right.$$

where *x* is the coordinate of particle position; *t* is the vibration time; *ƒ* is the input frequency; A is the amplitude; n is the number of standing waves; and *l* is the effective length of the string. The motion curve of the string was calculated using MATLAB 2018 software (Mathworks, Natick, MA, USA). All strings oscillate in parallel planes, as shown in Figure 2.

A 3D structural model of the string was built using SolidWorks 2020 (Solidworks, Waltham, MA, USA) according to actual dimensions. The electric field distribution in the spinning area was simulated with Ansoft Maxwell 3D in ANSYS Workbench 17.2 (ANSYS Corporation, Canonsburg, PA, USA). The software’s standard configurations were utilized for mesh generation and numerical simulation. The simulation parameters were set at the software’s default values. The string was set to the “phosphor bronze” material, while the receiver plate was designated as “copper”. The electric field strength was calculated by Equation (2) [25].

(2) E=−∇V

where *E* and *V* are the electric field strength and electric potential, respectively.

**Figure 3:**
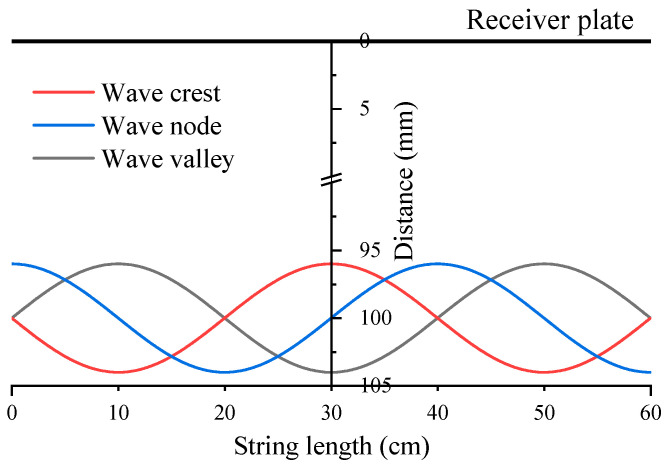
The position of data collection points.

Due to the fact that the center of the string was the concentrated region of the Taylor cone formation, the data points were collected in the vertical direction at the center of the string. The sampling points were taken starting from the receiving plate, moving vertically downward along the plane of the string (shown in Figure 3). Furthermore, the electric field distributions at the instances when the string was at the crest, valley, and node were compared.

### 2.5. Analytical Methods

The fiber morphology was determined using scanning electron microscopy (SEM) (JSM-7610F, JEOL Ltd. Tokyo, Japan). The diameters of the fibers in the SEM images were measured using Image-Pro 6.0 software, and the average value was reported. The fiber productivity was determined using the gravimetric method. After the multi-string standing wave electrospinning apparatus operated continuously for 20 min, the fibers were collected from the receiver plate and weighed using an electronic balance (B3M220, Shanghai Zhuojing Electronic Technology Co., Shanghai, China).

## 3. Results and Discussions

### 3.1. Process Parameters Optimization Based on Electric Fields Simulations

#### 3.1.1. Parallel Spacing of Strings

**Figure 4:**
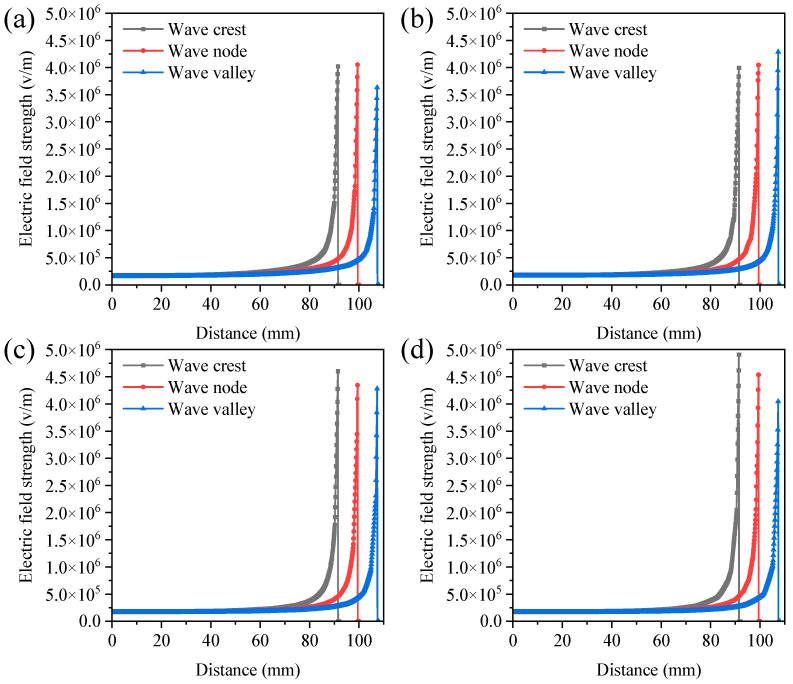
The electric field strength at various positions perpendicular to the string center points under the string spacings of 20 mm (**a**), 30 mm (**b**), 40 mm (**c**), and 50 mm (**d**). Simulation parameters: spinning voltage of 28 kV, spinning distance of 10 cm, standing wave number of 3, string number of 2, and phase difference in string standing wave of 0.

When multiple strings were used in the spinning process, the electric fields between the strings interacted with each other. If the string spacing is too narrow, electrostatic interference might occur, which decreases electrospinning process stability and nanofiber quality [26]. The electric field distribution was investigated through finite element simulation. Figure 4 and Figure S2 illustrate the electric field strength at various positions perpendicular to the string center points at the string spacings of 20, 30, 40, and 50 mm. Consistent with previous findings, the electric field strength near the string was stronger, facilitating the formation of the Taylor cone in the string droplet and enhancing fiber yields [24]. Strong electrostatic interference between the strings was observed when the string spacing was 20 mm and 30 mm. The electric field strength at the peaks, nodes, and troughs was relatively low. However, the electrostatic interference became minimal when the spacing was increased to 40 mm and 50 mm. Similarly, the maximum electric fields corresponding to string spacings of 40 mm and 50 mm were 1.60 × 10^7^ V/m and 1.67 × 10^7^ V/m, significantly higher than those at string spacings of 20 mm (1.36 × 10^7^ V/m) and 30 mm (1.48 × 10^7^ V/m) (Figure S3). The increased intensity of the electric field in the spinning zone enhanced the driving forces and enabled more effective stretching of jets, resulting in an improvement in the quality and production of nanofibers [25,27]. Compared to a single string (1.77 × 10^7^ V/m), the maximum electric field under two-string conditions was reduced by 5.65% to 23.16%, indicating that electrostatic interference was inevitable when strings were aligned in parallel. The greater the spacing between the strings, the smaller the electric field interference. When the spacing between the two strings was greater than 40 mm, the electric field variation was reduced to less than 10%. Thus, the optimal spacing between the strings was determined to be 40 mm.

#### 3.1.2. The Number of Strings

**Figure 5:**
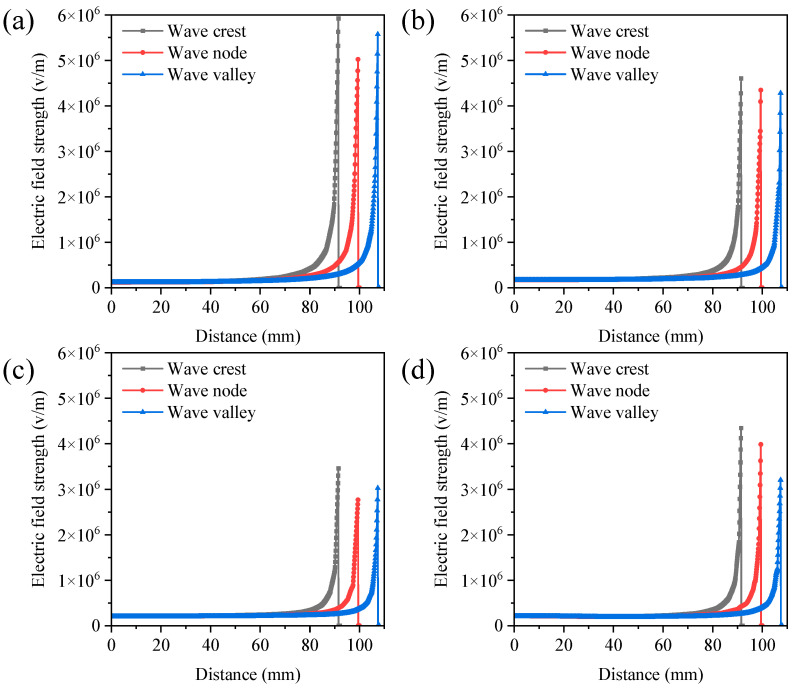
The electric field strength at various positions perpendicular to the string center points: (**a**) single string; (**b**) one side of double strings; (**c**) middle string of triple strings; (**d**) one side of triple strings. Simulation parameters: spinning voltage of 28 kV, spinning distance of 10 cm, standing wave number of 3, string spacing of 40 mm, and phase difference in string standing wave of 0.

The number of strings directly influences electrospinning production; typically, an increase in the number of strings results in higher spinning output. However, when the number of strings reaches or exceeds three, the side strings might cause electric field interference with the others, decreasing fiber quality [26]. To quantify the degree of interference from multiple strings on the electric field, the electric field distribution for one to three strings was investigated. Figure 5 and Figure S4 present the effect of the number of strings on the electric field distribution. Different string arrangements resulted in variations in the spatial electric field intensity distribution. Compared to a single string, the arrangement of two and three strings led to a certain degree of electric field weakening. Furthermore, in the case of three strings, the electric field intensity of the middle string was significantly lower than that of the side strings. When the electric field strength is insufficient, it might be difficult for the polymer solution to form a stable Taylor cone [28]. As a result, the polymer solution emitted from the nozzle fails to be sufficiently stretched, leading to issues such as increased fiber diameter and reduced uniformity [26]. The maximum electric field intensity in the spinning area also varied with the number of strings. For one, two, and three strings, the maximum spatial electric field intensities were 1.77 × 10^7^ V/m, 1.60 × 10^7^ V/m, and 1.58 × 10^7^ V/m, respectively (Figure S5). Considering that the electric field decreased by 10% when the number of strings increased to three, the optimal number of strings was determined to be two.

#### 3.1.3. Phase Difference in String Standing Waves

**Figure 6:**
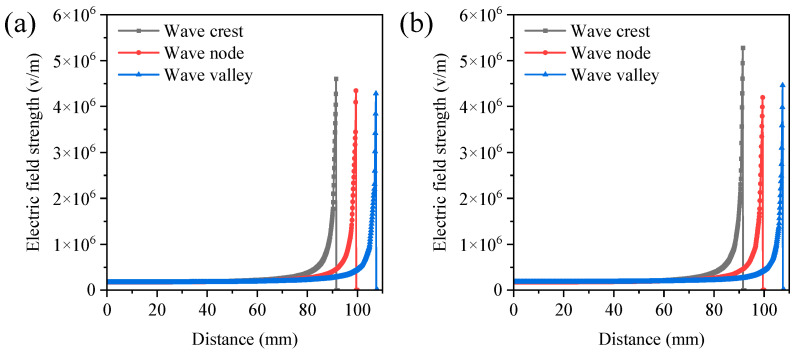
The electric field strength at various positions perpendicular to the string center points under the conditions of zero-phase difference (**a**) and half a period (**b**). Simulation parameters: spinning voltage of 28 kV, spinning distance of 10 cm, standing wave number of 3, string number of 2, and string spacing of 40 mm.

When there was a phase difference between two strings, the spatial distance between them increased, significantly affecting the electric field distribution. Therefore, it was imperative to study the impact of the phase difference in standing waves on the electric field distribution. The electric field distributions of two strings with phase differences of 0 and half a period were investigated using finite element electric field simulations. Under the condition of a half-period phase difference, the electric field intensity at the positions of the wave peaks and troughs was significantly higher than in the case of a 0-phase difference (Figure 6 and Figure S6). This phenomenon might be due to the fact that when one string was at the wave peak, the other string was at the wave trough, significantly increasing the spatial distance between the two strings and weakening the electric field interference. When the phase difference was 0, the maximum electric field intensity in the spinning area was 1.60 × 10^7^ V/m. While under the half-period phase difference condition, the maximum electric field intensity in the spinning area reached 1.77 × 10^7^ V/m, the same as that of a single string (Figure S7). These results indicated that adjusting the phase difference in the strings to half a period could effectively reduce the degree of electric field interference between the two strings. Thus, the optimal phase difference in string standing waves was determined to be one-half of a period.

### 3.2. Nanofiber Quality and Production of Multi-String Standing Wave Electrospinning

**Figure 7:**
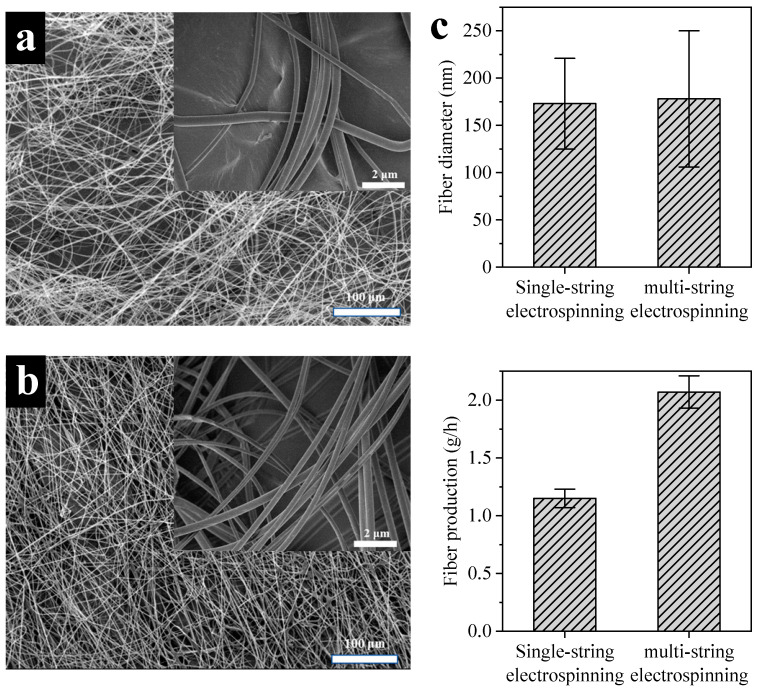
The fiber morphology and production under single-string and multi-string conditions: (**a**) the morphology of fibers from single-string electrospinning; (**b**) the morphology of fibers from multi-string electrospinning; (**c**) comparison of fiber diameter and production. The single-string electrospinning conditions: spinning voltage of 28 kV, spinning distance of 10 cm, and standing wave number of 3. The multi-string electrospinning conditions: spinning voltage of 28 kV, spinning distance of 10 cm, standing wave number of 3, string spacing of 40 cm, string number of 2, and phase difference of half a period between the standing waves on the strings.

Based on the discussions mentioned above, the optimal multi-string standing wave electrospinning process conditions were determined: a parallel spacing of 40 cm, the use of two strings, and a phase difference of half a period between the standing waves on the strings. Furthermore, the fiber quality and production between single-string and multi-string electrospinning were compared. Figure 7 shows the fiber morphology and production under single-string and multi-string conditions. As observed in Figure 7, the fiber morphology under both single-string and multi-string conditions was essentially similar, with comparable fiber diameters averaging 173 ± 48 nm and 178 ± 72 nm, respectively. However, transitioning from a single-string design to a multi-string design resulted in an increase in fiber production from 1.15 ± 0.08 g/h to 2.17 ± 0.14 g/h. In comparison to previously reported wire-type differential electrospinning (Table 1), the multi-string standing wave electrospinning proposed in this study had smaller average fiber diameters and low spinning voltage. Meanwhile, the fiber productivity exceeded that of most needleless electrospinning methods. The multi-string standing wave electrospinning exhibited great potential for the mass production of nanofibers.

## 4. Conclusions

A multi-string standing wave electrospinning apparatus was constructed in this study by installing a string array in the standing wave electrospinning device. The process parameters, including string spacing, quantity, and phase difference, were optimized, and their effects on the electric field distribution within the spinning area were investigated using electric field simulations. When the string spacing exceeded 40 mm, the number of strings was less than three, or the string standing wave phase difference was set to half a period, the electric field interference between strings could be effectively eliminated. The optimal string array parameters were determined to be a string spacing of 40 mm, two strings, and a phase difference of half a period. The multi-string standing wave electrospinning achieved a spinning diameter of 178 ± 72 nm, similar to that of single-string standing wave electrospinning (173 ± 48 nm). Still, it increased fiber production by 88.7%, reaching 2.17 g/h, thereby demonstrating the potential for the mass production of nanofibers. This work further refined the standing wave electrospinning process and provided a reference for optimizing wire-type electrospinning processes.

## Figures and Tables

**Table 1:** Comparison of different needleless electrospinning methods.

Spinning Method	Spinning Solution	Fiber Diameter	Spinning Voltage	Fiber Production	Reference
Pyramid-shaped spinneret electrospinning	PA-6	205 ± 84 nm	55 kV	2.30 g/h	[29]
Conical wire coil electrospinning	PVA	327 ± 123 nm	45 kV	0.86 g/h	[21]
Edge electrospinning	PCL	344 ± 67 nm	55 kV	0.68 g/h	[30]
Curved convex slot electrospinning	PVA	556 nm	50 kV	0.76 g/h	[23]
Spiral coil electrospinning	PVA	300 ± 110 nm	45 kV	2.94 g/h	[31]
Sprocket wheel disk electrospinning	PVA	426 ± 89 nm	50 kV	2.09 g/h	[32]
Embedded wire loop electrospinning	PAN	126 ± 79 nm	28 kV	0.48 g/h	[33]
Straight wire electrospinning	PVAc	1.21 μm	40 kV	/	[34]
Wire spinneret electrospinning	PVP	1.3 μm	40 kV	0.34 g/h	[35]
Linear flume electrospinning	PAN	171 ± 6.5 nm	60 kV	/	[36]
Multi-string standing wave electrospinning	PVA	178 ± 72 nm	28 kV	2.17 g/h	This study

## Data Availability

The original contributions presented in the study are included in the article/Supplementary Material, further inquiries can be directed to the corresponding authors.

## Supplementary Materials

The following supporting information can be downloaded at https://www.mdpi.com/article/10.3390/polym16162330/s1: Figure S1: The photograph during the experimental process. Figure S2: Partial enlargement of Figure 4. The electric field strength at various positions perpendicular to the string center points under the string spacings of 20 mm (a), 30 mm (b), 40 mm (c), and 50 mm (d). Simulation parameters: spinning voltage of 28 kV, spinning distance of 10 cm, standing wave number of 3, string number of two, phase difference of string standing wave of 0. Figure S3: Distribution of maximum electric field intensity in the spinning area with different string spacing. Figure S4: Partial enlargement of Figure 5. The electric field strength at various positions perpendicular to the string center points: (a) single string; (b) one side of double strings; (c) middle string of triple strings; (d) one side of triple strings. Simulation parameters: spinning voltage of 28 kV, spinning distance of 10 cm, standing wave number of 3, string spacing of 40 mm, phase difference of string standing wave of 0. Figure S5: The maximum electric field intensity in the spinning area with different string numbers. Figure S6: Partial enlargement of Figure 6. The electric field strength at various positions perpendicular to the string center points under the conditions of zero phase difference (a) and half a period (b). Simulation parameters: spinning voltage of 28 kV, spinning distance of 10 cm, standing wave number of 3, string number of two, string spacing of 40 mm. Figure S7: The maximum electric field intensity in the spinning area under the conditions of zero phase difference and half a period.
