# Supplementary material for: Optimization Design of a Multi-String Standing Wave Electrospinning Apparatus Based on Electric Field Simulations
Source: Polymers (Basel). 2024 Aug 17;16(16):2330. doi: 10.3390/polym16162330 (PMC11360486; doi:10.3390/polym16162330)
Supplement: Supplementary file 1 [file polymers-16-02330-s001.zip › polymers-3151013-supplementary.pdf]

Supporting information

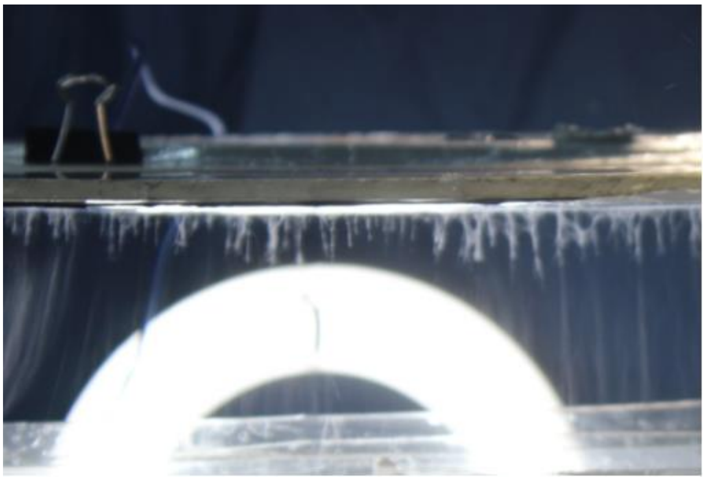

Figure S1. The photograph during the experimental process

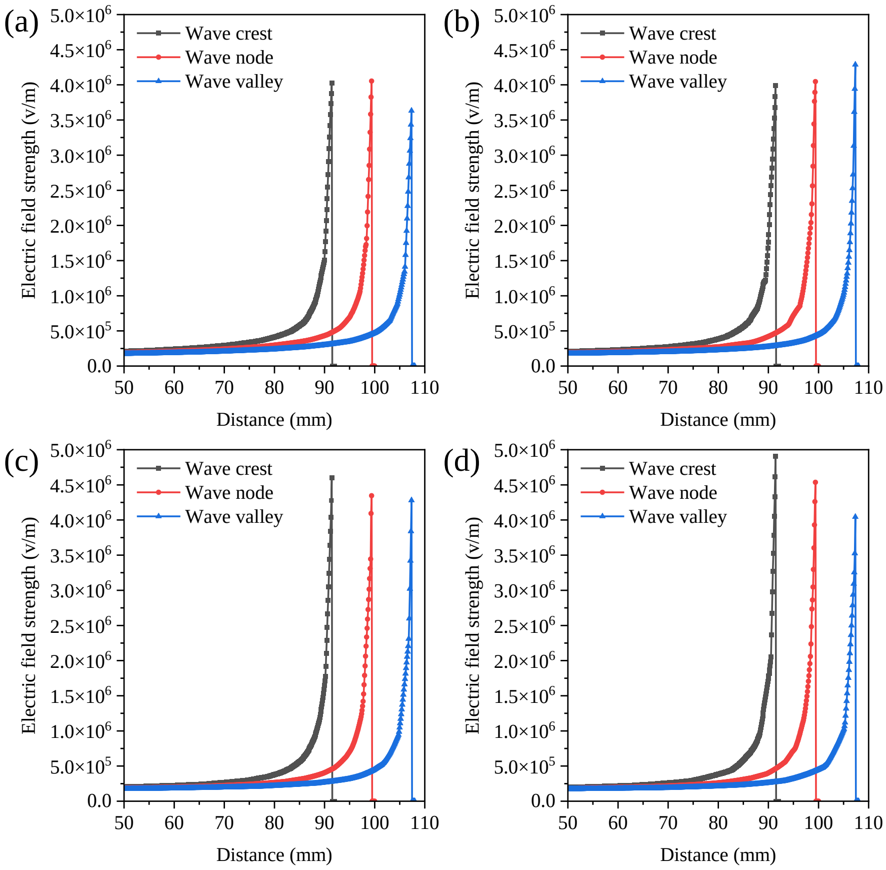

Figure S2. Partial enlargement of Figure 4. The electric field strength at various positions perpendicular to the string center points under the string spacings of 20 mm (a), 30 mm (b), 40 mm (c), and 50 mm (d). Simulation parameters: spinning voltage of 28 kV, spinning distance of 10 cm, standing wave number of 3, string number of two, phase difference of string standing wave of 0.

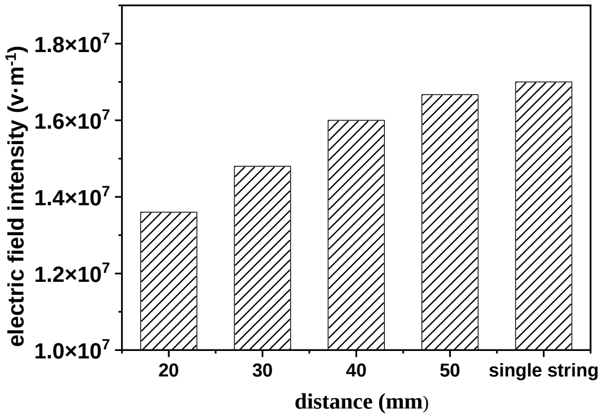

Figure S3. The maximum electric field intensity in the spinning area with different string spacing.

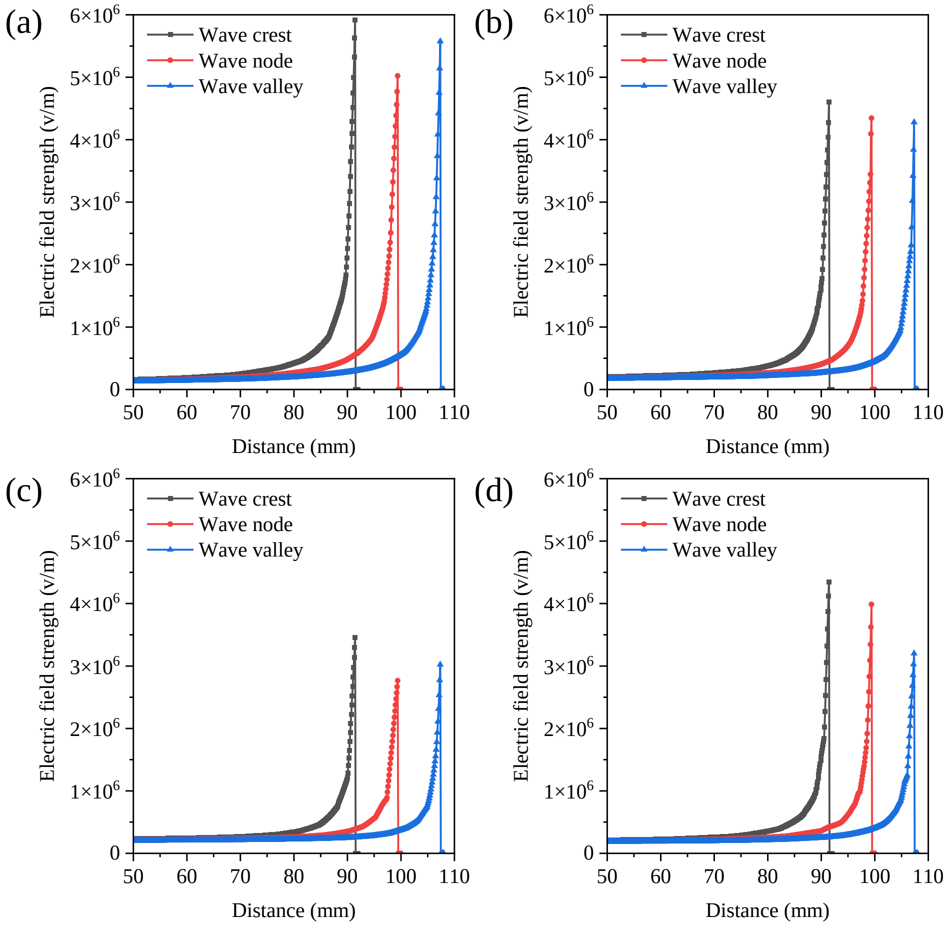

Figure S4. Partial enlargement of Figure 5. The electric field strength at various positions perpendicular to the string center points: (a) single string; (b) one side of double strings; (c) middle string of triple strings; (d) one side of triple strings. Simulation parameters: spinning voltage of 28 kV, spinning distance of 10 cm, standing wave number of 3, string spacing of 40 mm, phase difference of string standing wave of 0.

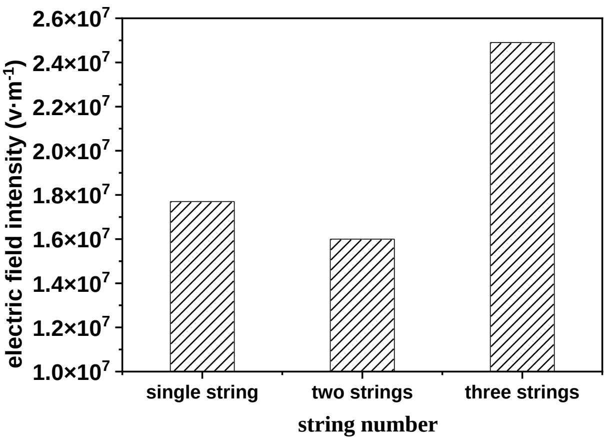

Figure S5. The maximum electric field intensity in the spinning area with different string numbers.

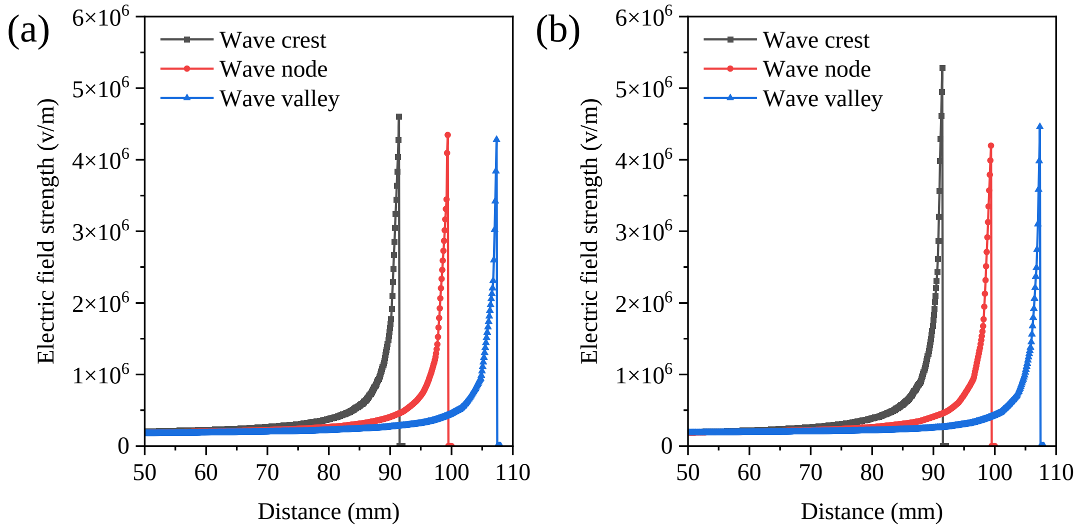

Figure S6. Partial enlargement of Figure 6. The electric field strength at various positions perpendicular to the string center points under the conditions of zero phase difference (a) and half a period (b). Simulation parameters: spinning voltage of 28 kV, spinning distance of 10 cm, standing wave number of 3, string number of two, string spacing of 40 mm.

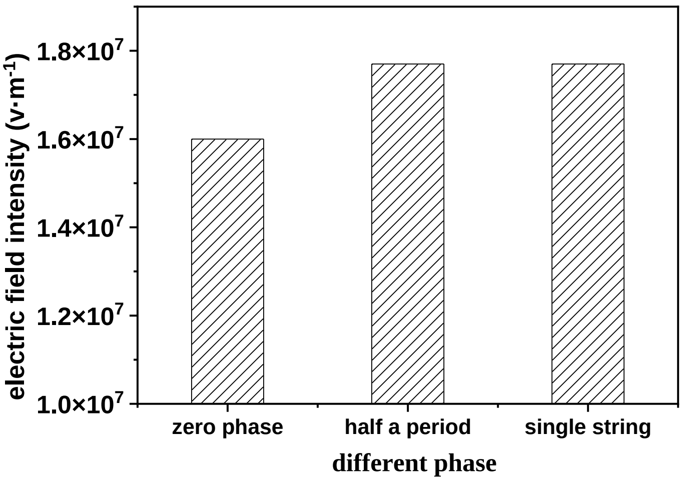

Figure S7. The maximum electric field intensity in the spinning area under the conditions of zero phase difference and half a period.
